# Microhomology-Mediated Mechanisms Underlie Non-Recurrent Disease-Causing Microdeletions of the *FOXL2* Gene or Its Regulatory Domain

**DOI:** 10.1371/journal.pgen.1003358

**Published:** 2013-03-14

**Authors:** Hannah Verdin, Barbara D'haene, Diane Beysen, Yana Novikova, Björn Menten, Tom Sante, Pablo Lapunzina, Julian Nevado, Claudia M. B. Carvalho, James R. Lupski, Elfride De Baere

**Affiliations:** 1 Center for Medical Genetics, Ghent University, Ghent, Belgium; 2 Department of Pediatrics, Ghent University Hospital, Ghent, Belgium; 3 Instituto de Genética Médica y Molecular (INGEMM), Hospital Universitario La Paz, Universidad Autónoma de Madrid, Madrid, Spain; 4 Department of Molecular and Human Genetics, Baylor College of Medicine, Houston, Texas, United States of America; University of Pennsylvania, United States of America

## Abstract

Genomic disorders are often caused by recurrent copy number variations (CNVs), with nonallelic homologous recombination (NAHR) as the underlying mechanism. Recently, several microhomology-mediated repair mechanisms—such as microhomology-mediated end-joining (MMEJ), fork stalling and template switching (FoSTeS), microhomology-mediated break-induced replication (MMBIR), serial replication slippage (SRS), and break-induced SRS (BISRS)—were described in the etiology of non-recurrent CNVs in human disease. In addition, their formation may be stimulated by genomic architectural features. It is, however, largely unexplored to what extent these mechanisms contribute to rare, locus-specific pathogenic CNVs. Here, fine-mapping of 42 microdeletions of the *FOXL2* locus, encompassing *FOXL2* (32) or its regulatory domain (10), serves as a model for rare, locus-specific CNVs implicated in genetic disease. These deletions lead to blepharophimosis syndrome (BPES), a developmental condition affecting the eyelids and the ovary. For breakpoint mapping we used targeted array-based comparative genomic hybridization (aCGH), quantitative PCR (qPCR), long-range PCR, and Sanger sequencing of the junction products. Microhomology, ranging from 1 bp to 66 bp, was found in 91.7% of 24 characterized breakpoint junctions, being significantly enriched in comparison with a random control sample. Our results show that microhomology-mediated repair mechanisms underlie at least 50% of these microdeletions. Moreover, genomic architectural features, like sequence motifs, non-B DNA conformations, and repetitive elements, were found in all breakpoint regions. In conclusion, the majority of these microdeletions result from microhomology-mediated mechanisms like MMEJ, FoSTeS, MMBIR, SRS, or BISRS. Moreover, we hypothesize that the genomic architecture might drive their formation by increasing the susceptibility for DNA breakage or promote replication fork stalling. Finally, our locus-centered study, elucidating the etiology of a large set of rare microdeletions involved in a monogenic disorder, can serve as a model for other clustered, non-recurrent microdeletions in genetic disease.

## Author Summary

Genomic disorder is a general term describing conditions caused by genomic aberrations leading to a copy number change of one or more genes. Copy number changes with the same length and clustered breakpoints for a group of patients with the same disorder are named recurrent rearrangements. These originate mostly from a well-studied mechanism, namely nonallelic homologous recombination (NAHR). In contrast, non-recurrent rearrangements vary in size, have scattered breakpoints, and can originate from several different mechanisms that are not fully understood. Here we tried to gain further insight into the extent to which these mechanisms contribute to non-recurrent rearrangements and into the possible role of the surrounding genomic architecture. To this end, we investigated a unique group of patients with non-recurrent deletions of the *FOXL2* region causing blepharophimosis syndrome. We observed that the majority of these deletions can result from several mechanisms mediated by microhomology. Furthermore, our data suggest that rare pathogenic microdeletions do not occur at random genome sequences, but are possibly guided by the surrounding genomic architecture. Finally, our study, elucidating the etiology of a unique cohort of locus-specific microdeletions implicated in genetic disease, can serve as a model for the formation of genomic aberrations in other genetic disorders.

## Introduction

Copy number variations (CNVs) are defined as DNA segments that are present at a variable copy number in comparison with a reference genome such as a deletions, duplications or insertions [1], [2]. In recent years it has become clear that CNVs are a major source of genetic diversity, competing with the single nucleotide variants (SNVs) as the main source of genetic variation between individuals. With the use of several technologies such as array-based comparative genomic hybridization (aCGH), single nucleotide polymorphism (SNP) genotyping and next-generation sequencing, numerous CNVs have been identified during the last decade [3]–[11]. Many of the identified CNVs represent benign polymorphic variants; however, CNVs can lead to a genetic disease when for instance a dosage-sensitive gene is affected. Such genetic diseases caused by genomic rearrangements are defined as genomic disorders [12]–[15]. The genomic rearrangements causing these disorders can be recurrent sharing a common interval and size, and having clustered breakpoints in multiple different subjects. These rearrangements are mostly the result of nonallelic homologous recombination (NAHR) between low-copy repeats (LCRs) or segmental duplications (SDs), a recombination-based mechanism [16]. In contrast, non-recurrent, locus-specific rearrangements can vary in size and have scattered breakpoints, thus suggesting the absence of a recombination hotspot. Only recently, several mechanisms causing non-recurrent genomic rearrangements have been proposed such as (i) non-replicative repair mechanisms: non-homologous end-joining (NHEJ) [17], microhomology mediated end-joining (MMEJ) [18] and NAHR between repetitive elements (for example, *Alu* or L1) [19], [20]; and (ii) replicative-based repair mechanisms: fork stalling and template switching (FoSTeS) [21], microhomology-mediated break-induced replication (MMBIR) [22], serial replication slippage (SRS) [23] and break-induced SRS (BISRS) [24]. Interestingly, as genomic rearrangements are assumed not to be random events, it has been proposed that the local genomic architecture other than LCRs or SDs stimulates these mechanisms by predisposing to CNV formation [25]. Indeed, several studies have revealed repetitive elements, sequence motifs or non-B DNA conformations overlapping with or located in the vicinity of CNV breakpoints. Another genomic characteristic frequently observed at the junctions is microhomology. These studies confirm that the majority of non-recurrent, locus-specific, pathogenic CNVs are not caused by NAHR, but rather by a diverse range of mechanisms [26]–[35]. The conclusions of these studies are however mostly based on a small number of sequenced junctions. Therefore, it was our aim to investigate which mechanisms underlie a large, unique set of locus-specific non-recurrent genomic rearrangements causing the rare developmental disorder blepharophimosis-ptosis-epicanthus inversus syndrome (BPES) [MIM #110100]. This disorder is characterized by a complex eyelid malformation with or without ovarian dysfunction [36], [37]. BPES is an autosomal dominant disorder caused by genetic defects of the *FOXL2* locus [38]–[44]. Even though intragenic mutations are most prevalent (81%), an important fraction of BPES cases is caused by heterozygous deletions. These deletions can encompass the *FOXL2* gene (12%) or can be located outside the *FOXL2* transcription unit removing potential regulatory elements such as conserved non-coding sequences (CNCs) and the long non-coding RNA (lncRNA) *PISRT1*, necessary for the correct transcription of *FOXL2* (5%) [41]–[44]. Here, we study 32 *FOXL2* encompassing and 10 regulatory deletions, respectively. As the observed deletions range from 1.4 kb to 5.51 Mb and the breakpoint locations are heterogeneous, a common deletion mechanism such as NAHR mediated by LCRs can be excluded. In order to unravel the underlying deletion mechanisms, we analyzed the extent of microhomology at the characterized breakpoints and explored the presence of repetitive elements, non-B DNA conformations and sequence motifs as well. We found that microhomology was present in 91.7% of 24 delineated breakpoint junctions. Moreover, particular genomic architectural features were found in all breakpoint regions. In conclusion, we propose that the majority of these deletions are caused by microhomology-mediated mechanisms such as MMEJ or the replicative-based repair mechanisms FoSTeS, MMBIR, SRS and BISRS. Finally, the genomic architecture might stimulate the formation of these rare deletions by increasing the susceptibility for DNA breakage or promote replication fork stalling.

## Results

### Delineation of the deletions

**Figure 1:**
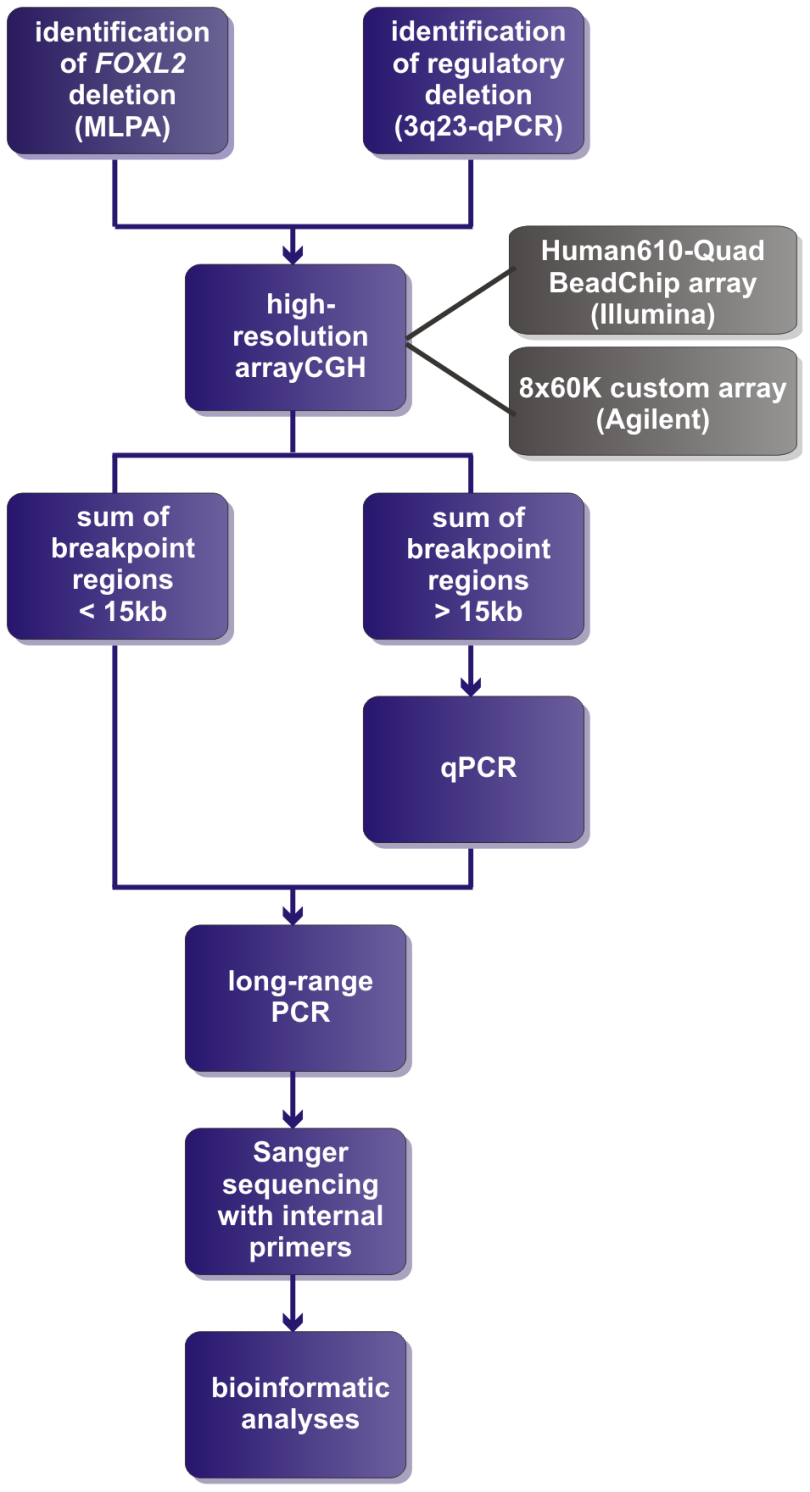
Delineation strategy. All *FOXL2* encompassing deletions were initially identified using MLPA. The regulatory deletions were identified using a combined approach of microsatellite analysis and a custom-made quantitative PCR assay of the *FOXL2* region (qPCR-3q23) [42]–[44]. For further delineation of the deletions two different array-based methods were used in a first step: (1) custom high-resolution 8×60 K Agilent microarrays for 35 deletions at the CMGG, and (2) genome-wide Illumina Human610-Quad BeadChip arrays for 7 deletions at the INGEMM. Subsequently, long-range PCR was performed if the sum of the breakpoint regions was smaller than 15 kb. However, if the sum of breakpoints was larger than 15 kb, the breakpoint regions were first further delineated using a qPCR-based copy number screening approach. Long-range PCRs resulting in a specific junction product underwent sequencing with internal primers. Finally, several bioinformatic tools were used in order to determine the underlying deletion mechanism.

**Figure 2:**
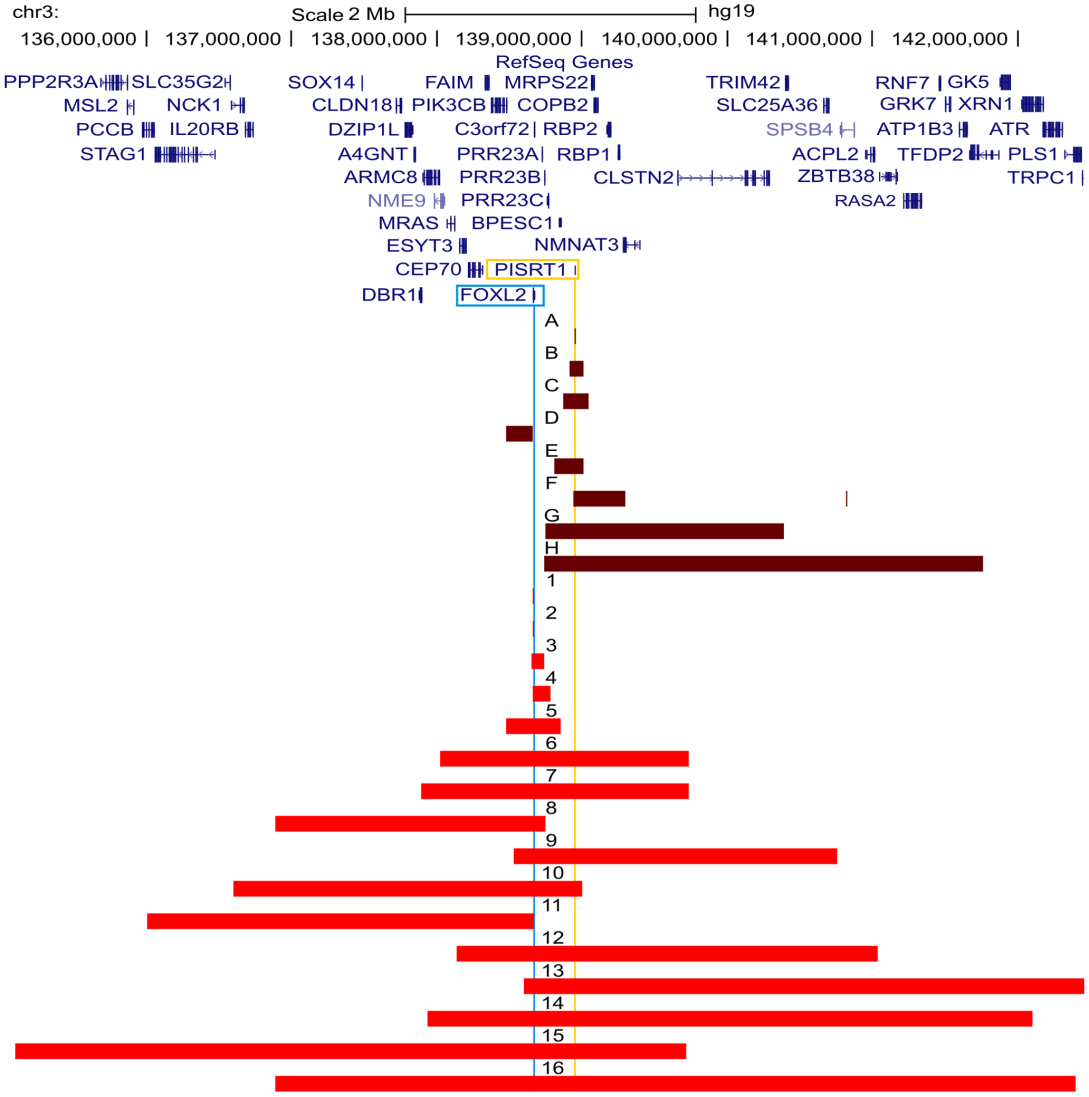
Overview of the delineated regulatory and *FOXL2* encompassing deletions. Overview of the *FOXL2* region (chr3:135099979–142458004; UCSC, Human Genome Browser, hg19) with custom tracks showing the delineated regulatory and *FOXL2* encompassing deletions presented in this study, numbered from A to H and from 1 to 16 respectively. The horizontal red bars indicate the deleted regions (regulatory deletions are shown in dark red and *FOXL2* encompassing deletions are shown in light red). At the top, the RefSeq Genes track is included. The locations of *FOXL2* and long non-coding RNA *PISRT1* are indicated by vertical blue and yellow lines respectively. Additional information on genes (i) contained in the deletion, (ii) spanning the breakpoints, or (iii) located outside the respective deletion and their distances to the breakpoint, can be found in Table S1.

Two of the 42 deletions were already delineated at base-pair resolution in previous studies [42], [43]. For the delineation of the remaining 40 deletions a strategy was followed as described in Figure 1. In short, a combination of aCGH, qPCR, long-range PCR and Sanger sequencing was applied. Based on the aCGH and qPCR analyses, long-range PCR was performed for 35 deletions of which 22 resulted in a specific junction product. The inability to obtain a product for the remaining 13 deletions may relate to the complexity of the genomic sequence at these junctions. To overcome this, several primer combinations were used however this was without success. The 22 specific junction products underwent Sanger sequencing to determine the exact physical location of the breakpoints. The *FOXL2* encompassing deletions ranged from 1.4 kb to 5.51 Mb while the regulatory deletions ranged from 7.4 kb to 3.02 Mb, including one complex deletion consisting of two deletions interspersed with a segment without copy number variation (namely deletion F, Figure S1). Overall, we were able to characterize the exact breakpoints of 16 *FOXL2* encompassing (1–16) and 8 regulatory deletions (A–H) using this strategy (Figure 2).

### Bioinformatic analyses

**Figure 3:**
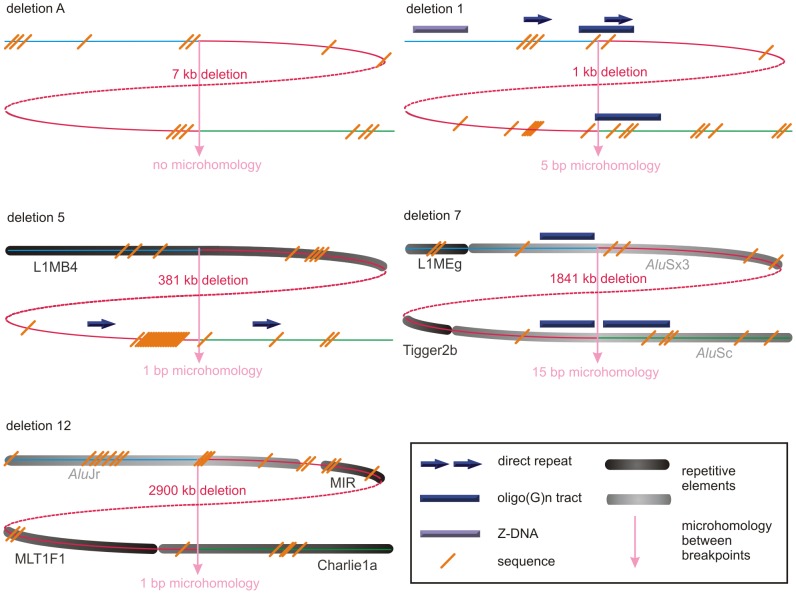
Schematic representations of the genomic architecture for 5 exemplary regulatory and *FOXL2*-encompassing deletion. For deletions A, 1, 5, 7 and 12, both breakpoint regions joined by the deletion are shown. These deletions were selected as an example for each group (group 1: deletion A, group 2: deletion 7, and group 3: deletions 1, 5 and 12) of most likely molecular mechanism as described in the discussion. A breakpoint region is displayed as the combination of two colored, solid lines together representing a 150 bp DNA sequence. The proximal breakpoint region consists of a non-deleted blue line and a deleted red line while the distal breakpoint region consists of a deleted red line and a non-deleted green line. Each deletion is composed of the two red, solid lines joined by the red dashed line which represents the different size of the deletion for every patient. The actual size of the deletions is indicated above the red, dotted lines. The pink vertical arrows mark the position of the breakpoints displaying the number of base pairs of microhomology between both breakpoint regions and the junction product (see also Figure 4 and Figure S3). The presence of repetitive elements is shown as bars of different shades of gray (*Alu* elements are shown in light grey bars, other repetitive elements are shown in dark grey bars). Sequence motifs are indicated with orange, skewed lines intersecting with the sequence. Direct repeats, oligo(G)_n_ tracts and Z-DNA are represented by dark purple arrows, dark purple bars and light purple bars respectively. The schematic representations for the other deletions can be found in the online supplement (Figure S2).

The breakpoints of the locus-specific, non-recurrent deletions were subjected to an extensive bioinformatic analysis to explore underlying mechanisms and to assess the contribution of the genomic architecture. To this end, we analyzed the extent of microhomology at the breakpoints and investigated the presence of repetitive elements, sequence motifs and non-B DNA conformations. An overview of the output of the different bioinformatic analyses can be found in Table 1. Visual representations of the breakpoint regions with the observed local genomic architecture of 5 selected deletions are shown in Figure 3 and of the remaining deletions in Figure S2.

**Table 1:** Overview of bioinformatic results.

						Proximal breakpoint region			Distal breakpoint region				
Patient code	Start (hg19)	End (hg19)	Size (kb)	Micro-homology (bp)	Insertion, deletion or substitution	Repetitive element	Number of sequence motifs	Number of non-B DNA conformation prediction motifs*	Repetitive element	Number of sequence motifs	Number of non-B DNA conformation prediction motifs*	Sequence identity between repetitive elements	Potential molecular mechanism
A	138949150	138956510	7	-	-	-	8	-	-	6	-	-	NHEJ
B	138912808	139012600	100	5	-	L1PA4	6	-	MIRb	10	-	-	Replicative§/MMEJ
C	138867472	139048942	181	1	-	L1ME3F	3	-	MIRc	8	-	-	Replicative§/NHEJ
D	138479902	138662725	183	2	-	-	4	-	-	7	-	-	Replicative§/NHEJ
E	138805920	139012140	206	1	TC>AA	L1M7	7	-	-	11	-	-	Replicative§/NHEJ
F	138938973	139294473	356	1	-	L1Med	30	-	-	3	-	-	Replicative§/NHEJ
G	138745991	140393036	1647	33	-	L1PA5	9	-	L1PA4	8	-	93%	NAHR/Replicative§/MMEJ
H	138741281	141762242	3021	12	-	*AluSz*	8	1	*AluSz*	17	-	83%	NAHR/Replicative§/MMEJ
1	138664845	138666255	1	5	-	-	6	3	-	15	1	-	Replicative§/MMEJ
2	138658319	138666527	8	4	-	-	5	1	-	5	1	-	Replicative§/NHEJ
3	138649686	138736058	86	1	C>A	-	12	1	-	5	-	-	Replicative§/NHEJ
4	138661542	138786728	125	1	-	-	-	-	L1PA2	8	-	-	Replicative§/NHEJ
5	138475828	138857284	381	1	-	L1MB4	7	-	-	40	1	-	Replicative§/NHEJ
6	138019964	139735421	1715	-	del(T)	Tigger15a	2	-	*AluSc*	6	1	-	NHEJ
7	137894385	139735424	1841	15	-	*AluSx3*	7	1	*AluSc*	6	2	85%	NAHR/Replicative§/MMEJ
8	136887871	138746237	1858	66	-	L1PA3	4	-	L1PA5	4	-	93%	NAHR/Replicative§/MMEJ
9	138532388	140753510	2221	15	-	*AluY*	8	-	*AluY*	14	1	85%	NAHR/Replicative§/MMEJ
10	136604806	139000361	2396	2	-	-	7	-	-	11	-	-	Replicative§/NHEJ
11	136007507	138671922	2664	34	-	*AluJo*	7	-	*AluSz*	10	1	77%	NAHR/Replicative§/MMEJ
12	138134298	141034621	2900	1	-	*AluJr*	16	-	*Charlie1a*	7	-	-	Replicative§/NHEJ
13	138602856	142458004	3855	26	-	*AluSp*	9	-	*AluSp*	12	-	89%	NAHR/Replicative§/MMEJ
14	137934887	142100431	4166	25	-	*AluSx3*	36	1	*AluSz6*	36	1	74%	NAHR/Replicative§/MMEJ
15	135099979	139713853	4614	1	-	LTR33A	3	-	-	7	-	-	Replicative§/NHEJ
16	136887730	142397685	5510	5	-	L1PA3	9	-	L1PA3	6	-	96%	NAHR/Replicative§/MMEJ

* : Non-B DNA conformations should be located at both sides of the junction or overlapping the junction.

§ : Replicative stands for replicative-based mechanisms and includes FoSTeS, MMBIR, SRS and BISRS.

### Microhomology

**Figure 4:**
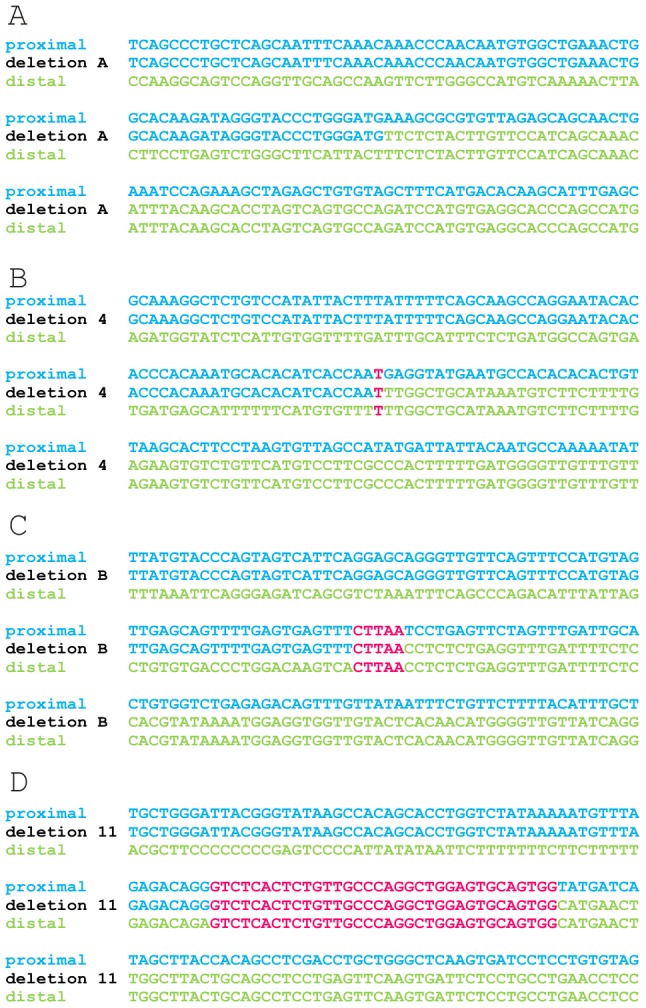
Multiple sequence alignment of 4 exemplary junctions. The junctions of deletion A (A), 4 (B), B (C) and 11 (D) are shown as an example for the different lengths of microhomology. Sequences of 150 bp surrounding each junction are aligned to the proximal and distal reference sequences using ClustalW. The proximal and distal reference sequences are shown in blue and green respectively. The junction sequences are depicted in the colour of the reference sequence they align with. Microhomology between the proximal and distal reference sequence and the junction are shown in pink. The other multiple sequence alignments can be found in the online supplement (Figure S3).

**Figure 5:**
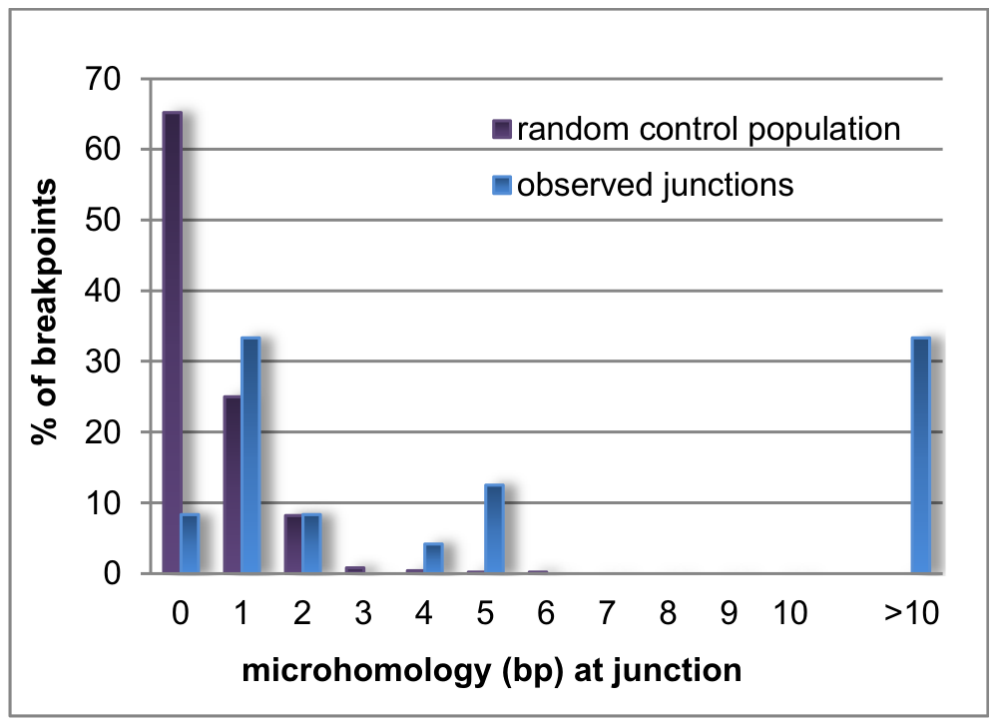
Distribution pattern of microhomology. Bar chart displaying the distribution of microhomology in the random control population (purple) and the observed breakpoints in this study (blue). Microhomology in the random control population clusters around 0 to 1 bp, while longer stretches of microhomology are noted for the observed breakpoints.

Microhomology is defined as one or more base pairs (bp) of perfectly matching sequence shared between the proximal and distal reference sequences surrounding the breakpoints. Also, it is an important hallmark of several mechanisms [14]. The extent of microhomology was evaluated using multiple sequence alignments (Figure 4, Figure S3). Of the 24 deletion junctions analyzed, 22 (91.7%) displayed microhomology between their breakpoints, ranging from 1 bp up to 66 bp. Only two deletions (deletion A and 6) showed a perfect transition at their junction of which one (deletion 6) was accompanied by a deletion of one bp. To exclude whether the observed microhomology at the breakpoints did just occur by chance, we compared our results against a random control population of 500 human genomic sequences representing artificial breakpoint regions. Using a Fisher's exact test we observed that microhomology is significantly enriched (p = 2.28×10^−08^) at our studied breakpoints. In addition, using a Wilcoxon rank sum test we observed that the distribution of microhomology in our breakpoints significantly differed (p = 2.21×10^−12^) from the random control population (Figure 5). This distribution pattern is in accordance with the ones observed by previous studies [45], [46].

### Repetitive elements

The Repeat Masker track in the UCSC genome browser was used to analyze the presence of known repetitive elements intersecting the breakpoints. A repetitive element was found at 31 of 48 breakpoints (64.6%) (Table 1). In the random control population a repetitive element was observed to intersect with 236 of 500 breakpoints (47.2%). Using a Fisher's exact test, we could conclude that our breakpoints are indeed significantly enriched with repetitive elements (p = 2.4×10^−2^). Interestingly, *Alu* elements were observed about three times more at our breakpoints in comparison with the control population (29.2% versus 10.6%). Indeed, when performing a Fisher's exact test with Bonferroni correction, we observed a significant enrichment of *Alu* elements at our breakpoints (p = 0.001). The frequency of L1-elements does not significantly differ from the control population (25% versus 16.2%; p = 0.156). In 13 of 24 deletions (54.2%), a repetitive element was observed at both breakpoints. Of these, 9 had repetitive elements belonging to the same class consisting of 6 *Alu*-*Alu* and 3 L1PA-L1PA combinations. In these cases, a Blast2 analysis was performed to determine the percentage of sequence identity between the repetitive elements. The highest percentage of sequence identity was observed between two L1PA3 elements in deletion 16 (96%). The lowest percentage of sequence identity was observed between an *Alu*Sx3 and an *Alu*Sz6 in deletion 14 (77%). The percentages for the other 7 deletions can be found in Table 1.

### Sequence motifs

The well-known capacity of sequence motifs to predispose to DNA breakage led us to analyze the nucleotide context of the breakpoint regions for the presence of 40 known sequence motifs [47]. An overview of the results can be found in Table S2. This analysis was also performed for the random control population. In total, 26 of 40 sequence motifs were present in one or more breakpoint regions. Only the proximal breakpoint region of deletion 4 did not contain a sequence motif. In comparison with the random control population, we observed that none of the motifs was significantly overrepresented in our breakpoint regions. In addition to individual motifs, we also analyzed if the overall density of sequence motifs might be increased. For this purpose, we counted the number of motifs present in each breakpoint region for the studied deletions and the random control population. In our deletions we observed a mean of 9.69 motifs per breakpoint region while a mean of 7.86 was observed for the random control population. However, the overall density of sequence motifs does not differ significantly (Wilcoxon rank sum test, p = 0.207). No new sequence motifs could be found in our deletion cohort.

### Non-B DNA conformations

Different bioinformatic tools were applied to determine the presence of sequences capable of forming non-B DNA conformations. Of note, genomic architecture resulting from DNA conformational changes, but not the primary sequence information, is crucial in these processes [48]. In total, a sequence capable of forming a non-B DNA structure could be identified in 14 of the 48 breakpoints (29.2%). Such sequences were identified in 107 of the 500 (21.4%) breakpoint regions of the random control population indicating that the frequency of sequences capable of forming a non-B DNA structure does not differ significantly between both populations (Fisher's exact test, p = 0.208). The comparison with the random control population was made for the individual non-B DNA conformations as well. The frequency of slipped hairpin structures and left-handed Z-DNA does not differ significantly from those observed in the control population (Fisher's exact test, p>0.05). However, for the tetraplex structures a significant overrepresentation could be observed (Fisher's exact test, p = 0.006).

Notably, four deletions have sequences capable of forming non-B DNA conformations present in both breakpoint regions (Table S3). Even more remarkable is that the non-B DNA conformations are from the same class in these deletions. Deletion 14 has a direct repeat in both breakpoint regions, while an oligo(G)_n_ tract is observed in both breakpoint regions of deletions 1, 2 and 7 respectively.

Interestingly, of the 14 breakpoint regions harboring a sequence capable of forming non-B DNA conformations, only 1 breakpoint region belonged to a regulatory deletion (deletion H). This means that such sequences are significantly overrepresented in the breakpoint regions of the *FOXL2* encompassing deletions (Fisher's exact test, p = 0.018).

## Discussion

### Microhomology-mediated mechanisms cause deletions of the *FOXL2* locus

Non-recurrent CNVs can be caused by a large spectrum of different mechanisms which can be grossly classified as non-replicative - (NAHR, NHEJ and MMEJ) or replicative-based repair mechanisms (FoSTeS, SRS, BISRS and MMBIR). If successful, the only reminder of a rearrangement is a unique breakpoint signature which can be used as the key to unraveling the underlying mechanism. NAHR causes rearrangements by misalignment and subsequent unequal cross-over between nonallelic sequences in meiosis or mitosis. For NAHR to occur, segments of a minimal length sharing extremely high similarity or sequence identity - named minimal efficient processing segments (MEPS) - between the homologous recombination substrates are required. These are mostly LCRs but can also be L1s, *Alu* elements or pseudogenes [49]. Breakpoints of rearrangements inferred by NAHR should therefore be intersected by these elements. NHEJ is utilized by human cells to repair two-ended, double stranded DNA breaks. NHEJ is characterized by two main features. First, NHEJ does not require the presence of substrates with extended homology but can be facilitated by the presence of microhomology (1–4 bp). Second, NHEJ can leave an ‘information scar’ at the joint point comprising of the loss or insertion of several random nucleotides [17]. An alternative pathway of NHEJ is called MMEJ. The difference between these two is that while the presence of microhomology is optional in NHEJ, it is a requirement for MMEJ to occur. Also, MMEJ uses longer stretches of microhomology (5–25 bp) than those used in NHEJ [50]. Two similar models, FoSTeS and SRS, were proposed to explain the sequence complexity sometimes seen at breakpoints. According to these models, the DNA replication fork can stall; the lagging strand consequently disengages from the original template, switches to another replication fork and then restarts DNA synthesis on the new fork by priming it via the microhomology between the switched template site and the original fork. Switching to a downstream replication fork would therefore result in a deletion, while upstream switching results in a duplication [21], [23]. Although both models share the same hypothesis of fork template switching, a difference can be observed. While the SRS model assumes that replication slippage occurs on closely adjacent sites and causes DNA rearrangements of small sizes, the FoSTeS model emphasizes that the template switch can occur over long distances (even 100 kb or megabase size) and therefore cause DNA rearrangements on a much larger scale [49]. Further molecular details of FoSTeS and SRS were extended in two more generalized models, namely MMBIR and BISRS. The major feature distinguishing these generalized models is that they are initiated by a single-end, double strand DNA break generated by a collapsed fork to expose a 3′ end that can be used to prime synthesis at a distant fork [22], [24]. All of these replicative-based repair mechanisms do not only cause complex rearrangements but can also form simple rearrangements where the evidence for sequence complexity has been removed during the rearrangement process. In addition, these mechanisms may be stimulated by the local genomic architecture. Consequently, the only option to elucidate the mechanism behind a CNV, is to delineate it at base-pair resolution and examine the sequence context of the breakpoints. Of our deletions of the *FOXL2* locus, 24 could be delineated at the base-pair level. Using several bioinformatics tools, we could examine the sequence context of these deletions, define their breakpoint signature and deduce the most likely underlying mechanism. Remarkably, no major differences were observed between the mechanisms underlying *FOXL2* encompassing and regulatory deletions. Based on the observed breakpoint signatures, the deletions could be classified in three different groups. The first small group contains only two deletions (deletion A and 6) both of which have a perfect transition at the junction. Additionally, the loss of a T nucleotide at the junction of deletion 6 represents an information scar pointing to NHEJ as potential mechanism. The 9 deletions of the second group are characterized by the presence of repetitive elements of the same family at both breakpoints (deletion G, H, 7, 8, 9, 11, 13, 14 and 16) which could indicate that NAHR has caused these deletions like observed in other studies [28], [30], [32], [34], [35]. An *Alu*-*Alu*-mediated NAHR might have resulted in 6 deletions while the other three deletions probably result from a L1-L1-mediated NAHR. However, the level of sequence identity is probably too low in most deletions for NAHR to occur. Three deletions do have a high percentage of sequence identity over a long length between L1 elements (Table 1). These L1 elements could therefore provide the MEPS required for efficient NAHR. On the other hand, microhomology ranging from 5 bp to 66 bp is observed at the junctions of these 9 deletions, suggesting that a replicative-based repair mechanism may have formed these deletions instead of NAHR [51]. It has also been suggested that repetitive elements may represent more difficult sequences to replicate leading to an increased chance of replication fork stalling or collapsing [46]. Alternatively, formation of secondary structures within or between repetitive elements may contribute to generate DSBs and further contribute to genomic instability involving those elements. So the presence of a repetitive element may initiate a replicative-based repair mechanism while the observed microhomology then facilitates the template switching and serves as the priming site in the second replication fork. The above assumptions are purely speculative though and further experimental evidence is needed to substantiate them. Another possible mechanism underlying these deletions is MMEJ which requires microhomology of 5 bp or more. It is however currently impossible to distinguish between replicative-based repair mechanisms and MMEJ, as they all share the breakpoint signature, namely microhomology at the junction. Nonetheless, because none of the junctions have an information scar, replicative-based repair mechanisms are favored. The 13 deletions of the third group (deletion B, C, D, E, F, 1, 2, 3, 4, 5, 10, 12 and 15) also have microhomology at their junction but as opposed to the second group they only have a repetitive element at one of their breakpoints or in case both breakpoints intersect with a repetitive element, the elements are from a different family. The microhomology in this third group ranges from 1 bp to 5 bp. Like the deletions of the second group, these 13 deletions also could have resulted from NHEJ, MMEJ or replicative-based repair mechanisms but again favoring the latter because no information scar was present at the junctions. Nonetheless, NHEJ or MMEJ could still have occurred, where a distinction can be made between both based on the length of microhomology. Microhomology of 1–4 bp may facilitate NHEJ (deletions C, D, E, F, 2, 3, 4, 5, 10, 12 and 15) [17] while longer microhomology stretches of 5 bp or more are used by MMEJ (deletions B and 1) [50]. Interestingly, a substitution of one and two nucleotides was observed near the junctions of deletion 3 and E respectively. None of these substitutions are described as a known SNP and they originate most likely as a side-effect of the underlying mechanism. The majority of these mechanisms are based on the occurrence of DSBs and the subsequent repair of these breaks for the formation of genomic rearrangements. It has been described that the repair polymerases recruited for these processes, are more prone to errors and thus may incorporate wrong bases during DNA synthesis [52], [53]. These mutations are referred to as break-repair-induced mutations [54].

In conclusion, in this set of junctions of non-recurrent, locus-specific deletions involving the *FOXL2* locus, we propose that the majority of deletions are caused by the microhomology-mediated mechanisms MMEJ, FoSTeS, MMBIR, SRS or BISRS. This conclusion is in accordance with the observations of the most recent similar locus-specific studies [31]–[35]. Moreover, microhomology is observed at the majority of sequenced junctions in both locus-specific and genome-wide benign or pathological CNVs supporting the role of replicative-based repair mechanisms in CNV formation [55]. Less recent studies conversely suggest NHEJ to be the major mechanism in causing non-recurrent deletions. These studies were however performed before replicative-based repair mechanisms were reported [26]–[30]. Interestingly, when revisiting the data of these studies, microhomology is observed at more than half of these junctions indicating that replicative-based repair mechanisms could potentially also occur (Table S4). Furthermore, based on our results we hypothesize that other unique, non-recurrent, clustered microdeletion cohorts [56]–[60] are potentially also caused by a variety of microhomology-mediated mechanisms such as MMEJ, FoSTeS, MMBIR, SRS and BISRS.

### Local genomic architecture stimulates formation of non-recurrent deletions

The role of genomic architectural features in the formation of recurrent CNVs is well established as flanking LCRs or SDs act as homologous recombination substrates for an NAHR or ectopic recombination event mediated by these homologous sequence substrates. However, the role of genome architecture in non-recurrent rearrangements is currently still unclear. Studies like ours therefore contribute to the elucidation of a potential role of the genomic architecture and help delineate what those potential features may be. The presence of repetitive elements, sequences forming non-B DNA conformations and sequence motifs may lead to genomic instability and subsequently genomic rearrangements by promoting the formation of DSBs or by stalling the replication [48], [61]–[64]. Such genomic architectural features were observed in all breakpoint regions but only repetitive elements within particular *Alu* elements were found to be significantly enriched. To investigate whether this enrichment was not a bias, we compared the fraction of *Alu* elements in the CNV region with that in chromosome 3 and in the entire genome. Indeed, the fraction of sequence length occupied by *Alu* elements in the region containing the deletions (chr3:129230494–148645311, hg19) is only 8.32% which is comparable to the fraction found for chromosome 3 (8.84%) and the human genome 10.6% [65]. Overall, this indicates that *Alu* elements do occur more frequently at the breakpoints compared to the genome average. Although this observation is in accordance with a similar study by Vissers et al. [46], the mechanistic significance of this is currently unknown. Oligo(G)_n_ tracts capable of forming tetraplex structures also displayed a significant overrepresentation in the breakpoint regions. Interestingly, both breakpoint regions of deletions 1, 2 and 7 display an oligo(G)_n_ tract while deletion 14 has direct repeats in both breakpoint regions which could indicate that 2 DSBs have occurred in these deletions, favoring NHEJ or MMEJ. Conversely, the presence of the non-B DNA conformations in these and the other deletions can cause collapsing of the replication fork. Replicative-based repair mechanisms can therefore not be ruled out. Interestingly, sequences capable of forming non-B DNA conformations were observed more frequently in the breakpoints of the *FOXL2* encompassing deletions than in those of the regulatory deletions suggesting that the genomic architecture differs between both types of deletions. This might explain the higher prevalence of deletions encompassing *FOXL2*.

### General conclusion

We propose that the majority of non-recurrent deletions of the *FOXL2* locus are caused by microhomology-mediated mechanisms like MMEJ, FoSTeS, MMBIR, SRS or BISRS. Finally, the genomic architecture might drive the formation of these rare, locus-specific deletions by increasing the susceptibility for DNA breakage or promote DNA replication fork stalling. The insights from our locus-centered study investigating a large set of breakpoint sequences from non-recurrent, gene encompassing and regulatory microdeletions causing monogenic disease, can therefore serve as a paradigm for other clustered, non-recurrent microdeletions involved in genetic disease.

## Methods

### Ethics statement

This study was conducted following the tenets of Helsinki and approved by the institutional review board (99/250).

### Patients

Forty-two consenting BPES patients with a *FOXL2* encompassing (32) or regulatory deletion (10) were enrolled in this study. All patients were clinically diagnosed with BPES based on the presence of minimal three out of the four typical BPES features. Patients can be subdivided based on the genetic center where they were molecularly diagnosed. The largest group of deletions was diagnosed at the Center for Medical Genetics at Ghent University (CMGG) in Belgium. This group contains 25 *FOXL2* encompassing deletions and 10 regulatory deletions. The second group of 7 *FOX2* encompassing deletions was diagnosed at the Instituto de Genética Médica y Molecular (INGEMM) at the Hospital Universitario La Paz in Spain. Molecular diagnosis of all *FOXL2* encompassing deletions was performed using a commercially available multiplex ligation-dependent probe amplification (MLPA) mix (P054, MRC-Holland, Amsterdam, the Netherlands) according to the manufacturer's instructions. The regulatory deletions located outside the *FOXL2* transcription unit were identified using a combined approach of microsatellite analysis and a custom-made quantitative PCR assay in the *FOXL2* region (qPCR-3q23) as previously described [42], [43].

### High-resolution aCGH

Two different array-based methods were used: (i) custom high-resolution 8×60 K Agilent microarrays at the CMGG, and (ii) genome-wide Illumina Human610-Quad BeadChip arrays at the INGEMM. The custom high-resolution 8×60 K Agilent microarray was designed using the online design tool eArray (Agilent Technologies), targeting a region of 10 Mb around *FOXL2* (chr3:133517310–143517310; UCSC, Human Genome Browser, hg19) consisting of 52,800 probes spaced at an average density of 200 bp. Hybridizations were performed according to manufacturer's instructions with minor modifications [66]. The results were subsequently visualized in arrayCGHbase [67]. The genome-wide Illumina Human610-Quad BeadChip arrays contain 620,901 tag SNPs and have an average resolution of 4.7 kb. Hybridization and subsequent data-analysis was performed as previously described [44]. The proximal and distal breakpoint regions were defined as the regions between the last proximal normal and first deleted probe proximally, and the last deleted and first distal normal probe, respectively.

### Quantitative PCR (qPCR)

If the sum of the breakpoint regions outsized the predefined, arbitrary threshold of 15 kb, qPCR was used to reduce the breakpoint regions, resulting in more suitable fragments for long-range PCR. Primers were designed equally throughout the breakpoint regions and subjected to a stringent *in silico* and *in vitro* validation according to previously described parameters. The qPCR primers that qualified were used in a qPCR-based copy number analysis as previously described [68]. In short, 7.5 µl qPCR reactions contained 3.75 µl 2× master mix (qPCR core kit for SYBR Green I, Eurogentec), 0.375 µl of each primer (5 µM working solution), 1 µl nuclease-free water and 2 µl template (10 ng/µl). The reactions were carried out on the LightCycler 480 Instrument II (Roche) using the following qPCR protocol: 10 min pre-incubation at 95°C followed by 45 cycles of 95°C for 10 s, 60°C for 45 s and 72°C for 1 s, next a dissociation run from 60 to 95°C and ending with a cooling step. Data-analysis was performed with qBasePlus software [69]. Two reference genes were used for normalization of the relative quantities and two positives controls with known copy number were used as a reference to calculate the copy numbers [68].

### Long-range PCR and sequencing of junction products

For the delineation of the deletions at nucleotide level, specific junction products need to be obtained. Therefore, inward-facing PCR primers were designed in the normal regions flanking the breakpoint regions. Long-range PCR reactions were performed in a total volume of 20 µl containing 1× iProof HF buffer, 200 µM of each dNTP, 0.5 µM of each primer, 0.4 units of iProof DNA-polymerase (Bio-Rad) and 100 ng of template DNA. The standard PCR protocol is defined as follows: 94°C for 2 min, 35 cycles of (94°C for 30 sec, Ta for 30 sec, 68°C for 1 min/kb), and a final extension of 72°C for 10 min with an optimized annealing temperature and extension time for each junction product. To evaluate the specificity of a junction product, a control sample of a healthy individual accompanied the deletion samples. After amplification, the PCR products were visualized using the LabChip GX with the DNA 5K assay kit (Caliper Life Sciences) if junction products are assumed to be smaller than 5 kb or using gel electrophoresis. Next, specific junction products were sequenced using internal primers with the BigDye Terminator v. 3.1 Cycle Sequencing Kit (Applied Biosystems). Sequencing reactions were then loaded on an Applied Biosystems Prism 3130 or 3730 genetic Analyzer.

### Bioinformatic analyses

The sequences generated from the internal primers were first aligned to the reference sequence (obtained from UCSC, hg19) with SeqScape v1.1 (Applied Biosystems) to visualize the junction. To determine the exact genomic location of the breakpoints, the proximal and distal sequences flanking the junction were loaded into the Blat tool provided by the UCSC browser [70]. If microhomology was present at the junction, the genomic location of the proximal breakpoint was defined as the last nucleotide adjacent to the microhomology-stretch and the genomic location of the distal breakpoint was defined as the first nucleotide adjacent to the microhomology-stretch. Breakpoints, breakpoint regions and junction fragments were subjected to an extensive bioinformatic analysis, with breakpoint region defined as a 150 bp fragment surrounding a breakpoint and junction fragment as a 150 bp fragment surrounding the junction, to assess the involvement of the genomic architecture in the origin of the deletions. First, the presence of microhomology at the breakpoints was analyzed with a multiple sequence alignment between the proximal and distal breakpoint regions, and the junction fragment using ClustalW [71]. Second, the presence of known repetitive elements intersecting the breakpoints was investigated using the Repeat Masker track in the UCSC genome browser [72]. In cases where both breakpoints of a deletion overlap with a repetitive element, BLAST2 was used to determine the percentage of sequence identity between the elements [73]. Third, the presence of DNA sequences leading to non-B DNA conformations in the breakpoint regions was examined with several different tools: GT-repeats (forming left-handed Z-DNA) with Zhunt online [74]; direct, inverted and mirror repeats capable (forming slipped hairpin, cruciform and triplex structures, respectively) with RepeatAround [75]; oligo(G)_n_ tracts (forming tetraplex structures) with QGRS [76]. Non-B DNA conformations were only included if both counterparts flanked the breakpoint. And fourth, the presence of previously described sequence motifs [47] was analyzed with Fuzznuc [77]. These results were compared against a random control population representing the human genome as described by Vissers et al. [46] and Hannes et al. [78], to assess the statistical significance of the presence of genomic architecture. This random control population consists of 500 human genomic sequences of 150 bp each, randomly extracted from Ensembl using an in-house developed script. These sequences represent artificial breakpoint regions with the breakpoint between nucleotides 75 and 76. The same bioinformatic analyses were performed on these 500 sequences. The nucleotides surrounding the artificial breakpoint were evaluated for the presence of microhomology and the artificial breakpoints were analyzed for the possible presence of intersecting repetitive elements. Finally, the entire breakpoint regions were evaluated for the presence of motifs or sequences capable of forming non-B DNA conformations. Fisher's exact tests were performed to verify if the presence of a genomic element in the deletion population differed significantly in comparison with the control population.

## Supporting Information

Figure S1 aCGH profile of complex deletion F visualized in arrayCGHbase. At the top, for reference, chromosome 3 is represented with a red rectangle indicating the location of the displayed array profile. At the bottom, the genomic position is shown in more detail. The red (loss), green (gain) and black (no change) dots represent log_2_-ratios of individual oligonucleotides. The largest deletion spans 0.36 Mb and the smaller deletion is 8 kb long. Both deletions are separated by a copy neutral region of 35 kb.
(TIF)

Figure S2 Schematic representation of the genomic architecture of the remaining regulatory and *FOXL2* encompassing deletions. For the remaining deletions, both breakpoint regions joined by the deletion are shown. A breakpoint region is displayed as the combination of two colored, solid lines together representing a 150 bp DNA sequence. The proximal breakpoint region consists of a non-deleted blue line and a deleted red line while the distal breakpoint region consists of a deleted red line and a non-deleted green line. Each deletion is composed of the two red, solid lines joined by the red dashed line which represents the different size of the deletion for every patient. The actual size of the deletions is indicated above the red, dotted lines. The pink vertical arrows mark the position of the breakpoints displaying the number of base pairs of microhomology between both breakpoint regions and the junction product (see also Figure 4 and Figure S3). The presence of repetitive elements is shown as bars of different shades of gray (*Alu* elements are shown in light grey bars, other repetitive elements are shown in dark grey bars). Sequence motifs are indicated with orange, skewed lines intersecting with the sequence. Direct repeats, oligo(G)_n_ tracts and Z-DNA are represented by dark purple arrows, dark purple bars and light purple bars respectively.
(PDF)

Figure S3 Multiple sequence alignments. Sequences of 150 bp surrounding the junctions of each deletion were aligned to the proximal and distal reference sequences using ClustalW. The proximal and distal reference sequences are shown in blue and green respectively. The junction sequences are depicted in the colour of the reference sequence they align with. Microhomology between the proximal and distal reference sequence and the junction are shown in pink.
(PDF)

Table S1 The genomic location and gene content of the *FOXL2* encompassing and regulatory deletions.
(PDF)

Table S2 Overview of sequence motifs.
(PDF)

Table S3 Sequences of non-B DNA conformations.
(PDF)

Table S4 The presence of microhomology and the most likely molecular mechanism in previous studies.
(PDF)
