# Supplementary figures and images for: Microhomology-Mediated Mechanisms Underlie Non-Recurrent Disease-Causing Microdeletions of the FOXL2 Gene or Its Regulatory Domain
Source: PLoS Genet. 2013 Mar 14;9(3):e1003358. doi: 10.1371/journal.pgen.1003358 (PMC3597517; doi:10.1371/journal.pgen.1003358)

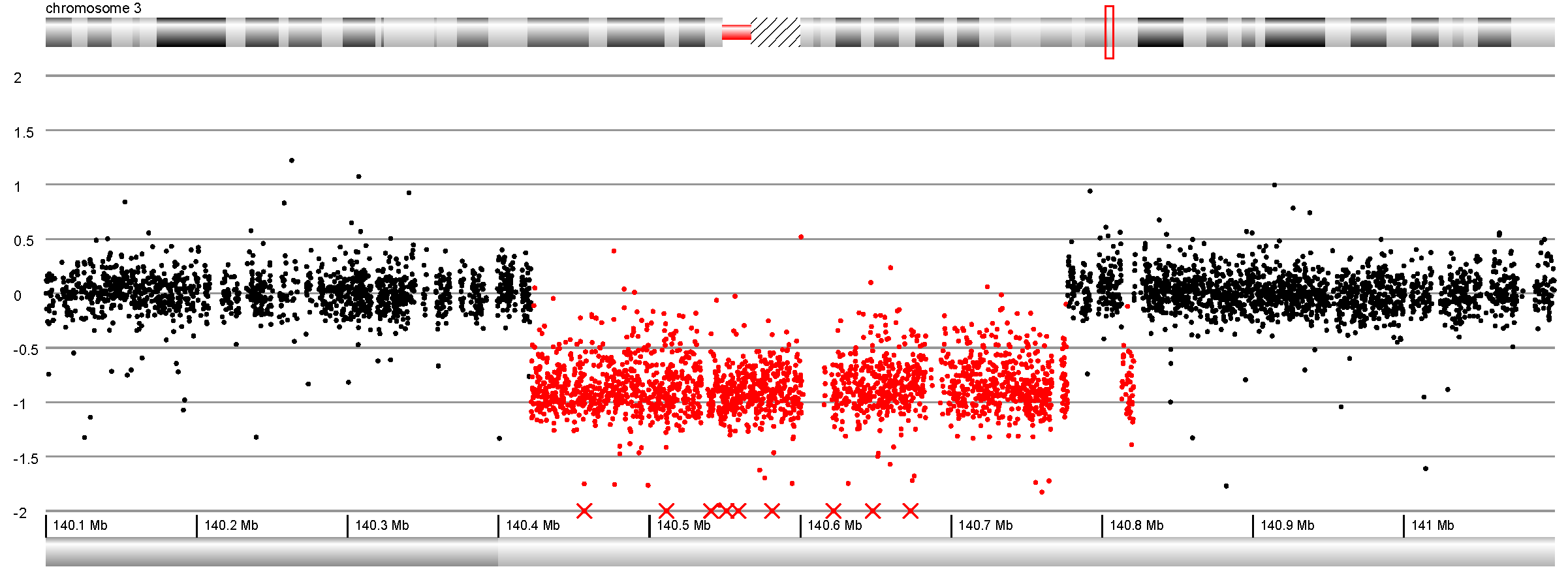

Supplement: Figure S1 — aCGH profile of complex deletion F visualized in arrayCGHbase. At the top, for reference, chromosome 3 is represented with a red rectangle indicating the location of the displayed array profile. At the bottom, the genomic position is shown in more detail. The red (loss), green (gain) and black (no change) dots represent log2-ratios of individual oligonucleotides. The largest deletion spans 0.36 Mb and the smaller deletion is 8 kb long. Both deletions are separated by a copy neutral region of 35 kb. (TIF) [file pgen.1003358.s001.tif]

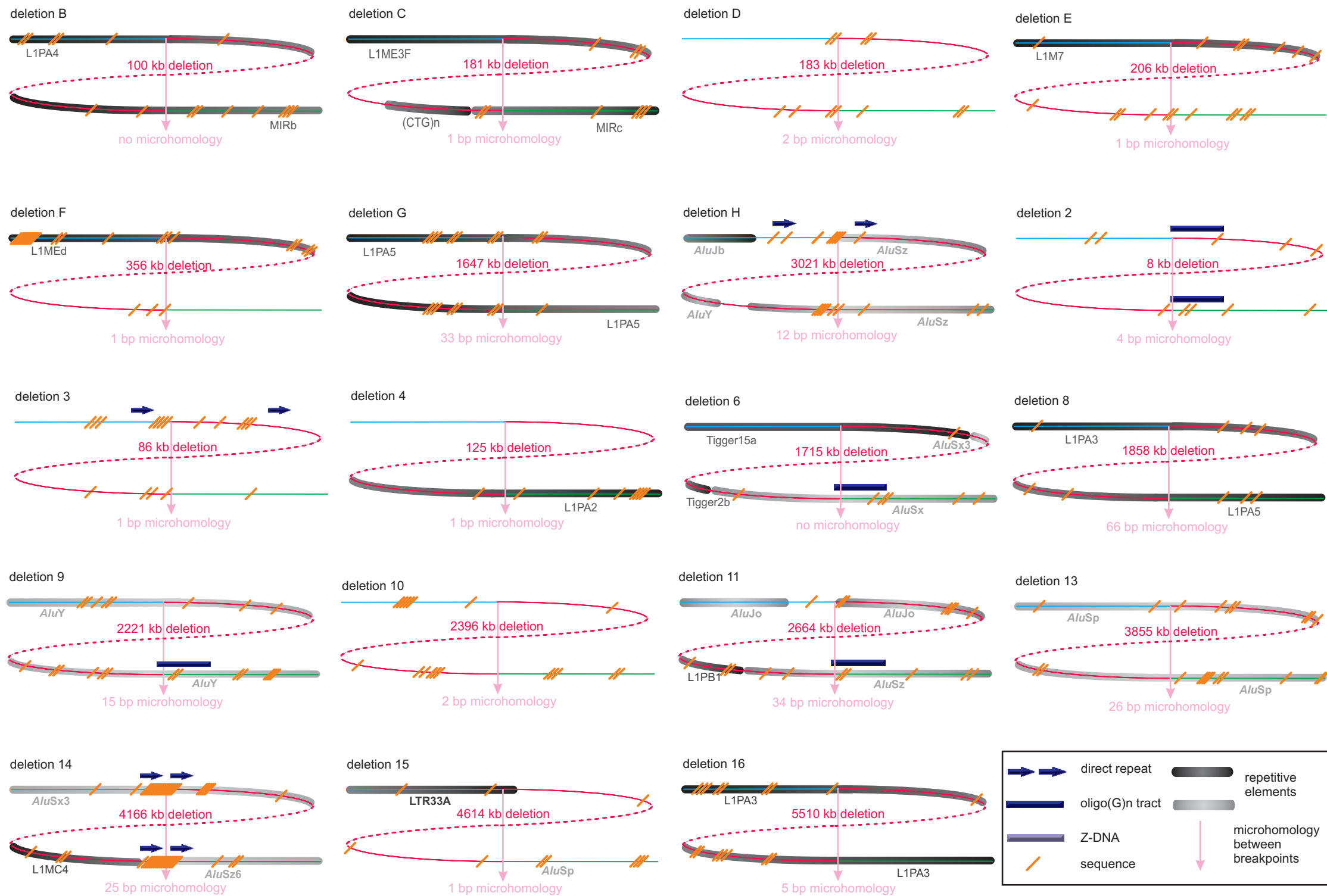

Supplement: Figure S2 — Schematic representation of the genomic architecture of the remaining regulatory and FOXL2 encompassing deletions. For the remaining deletions, both breakpoint regions joined by the deletion are shown. A breakpoint region is displayed as the combination of two colored, solid lines together representing a 150 bp DNA sequence. The proximal breakpoint region consists of a non-deleted blue line and a deleted red line while the distal breakpoint region consists of a deleted red line and a non-deleted green line. Each deletion is composed of the two red, solid lines joined by the red dashed line which represents the different size of the deletion for every patient. The actual size of the deletions is indicated above the red, dotted lines. The pink vertical arrows mark the position of the breakpoints displaying the number of base pairs of microhomology between both breakpoint regions and the junction product (see also Figure 4 and Figure S3). The presence of repetitive elements is shown as bars of different shades of gray (Alu elements are shown in light grey bars, other repetitive elements are shown in dark grey bars). Sequence motifs are indicated with orange, skewed lines intersecting with the sequence. Direct repeats, oligo(G)n tracts and Z-DNA are represented by dark purple arrows, dark purple bars and light purple bars respectively. (PDF) [file pgen.1003358.s002.pdf]
