# Supplementary material for: Microhomology-Mediated Mechanisms Underlie Non-Recurrent Disease-Causing Microdeletions of the FOXL2 Gene or Its Regulatory Domain
Source: PLoS Genet. 2013 Mar 14;9(3):e1003358. doi: 10.1371/journal.pgen.1003358 (PMC3597517; doi:10.1371/journal.pgen.1003358)
Supplement: Figure S3 — Multiple sequence alignments. Sequences of 150 bp surrounding the junctions of each deletion were aligned to the proximal and distal reference sequences using ClustalW. The proximal and distal reference sequences are shown in blue and green respectively. The junction sequences are depicted in the colour of the reference sequence they align with. Microhomology between the proximal and distal reference sequence and the junction are shown in pink. (PDF) [file pgen.1003358.s003.pdf]

proximal	AAATCAAATCCAGATGTGCTGTGTATCTAAATGAGAAATGTAAAACAATG
deletion C	AAATCAAATCCAGATGTGCTGTGTATCTAAATGAGAAATGTAAAACAATG
distal	ATTGCTATTATCATCACTGTATTCTTAGAGCTGCTGCTGCTGCTGCTGCA
proximal	AAACTTTACAAAGATAAGATAGGACAATAGCTTTATGACTTCGAGGGTAG
deletion C	AAACTTTACAAAGATAAGATAGGACACACAGATATGGGTTCAAGCCACTT
distal	GTGGAAGAACACAGCCCCTTCCAACACACAGATATGGGTTCAAGCCACTT
proximal	TGACAGTTTTTTATATAGCCCACAAATCCTGCTAAACATAAAGGAGAAGA
deletion C	GCCATTCAGCAGCTGTTAGGGCTTGGACAGGCCACATACCACCCCACAGC
distal	GCCACTCAGCAGCTGTTAGGGCTTGGACAGGCCACATACCACCCCACAGC
proximal	AATTCTGCAAGTGCTAATGAACTAACAGATCTAGGCAATGATCATTAATA
deletion D	AATTCTGCAAGTGCTAATGAACTAACAGATCTAGGCAATGATCATTAATA
distal	TCTATTAATGTTTGGAACAAACTCTTGGCTGTTATAGTTGACTCAAAATC
proximal	GATGCTAAAGACATAAATAATAATAAGGTGAAAGGAGAAGAAACTTTACA
deletion D	GATGCTAAAGACATAAATAATAATAACACAGCCAGCCCATACACCTAGTA
distal	GAAAAGTTTGAGAACAATAATAAAAACACAGCCAGCCCATACACCTAGTA
proximal	ATCGTCCGTGTCTGTCTTGTTGACACTACAGGAATCCCCTGATCTTAGCA
deletion D	CAACTGTGTATGAGGTAGAATACAAACTTGAATAGGTACAAAAATAAACC
distal	CAACTGTGTATGAGGTAGAATACAAACTTGAATAGGTACAAAAATAAACC
proximal	ATTTATTTTCCTTCACAATTTTAATCTTCTTTGCATTAATATTTCTCTTA
deletion E	ATTTATTTTCCTTCACAATTTTAATCTTCTTTGCATTAATATTTCTCTTA
distal	ACCAAGGGCACAAGTGACAGACGTTTCTGGCTTCTCTGTGTAGTTGGAGC
proximal	AGTATATTTGTTGTCTGCCATCTGTTTTTCTCATTTGTTTCTTGTAGCAT
deletion E	AGTATATTTGTTGTCTGCCAAATGTCCAGGCTGGATCATCCAGGATGGTT
distal	AGTCAGTGTGGCAGCCTCAGCCGCTCCAGGCTGGATCATCCAGGATGGTT
proximal	CTTTGAATAGTTCCTGTGCTGATTCCTTTTAAATTATTCATCTTTGAAGA
deletion E	GAAGCCTGAAGCCTGGGGGTAGAGGGAGGGGGCAGCCGTGAAATAAAAGG
distal	GAAGCCTGAAGCCTGGGGGTAGAGGGAGGGGGCAGCCGTGAAATAAAAGG
proximal	TAACTTTTTTTTTTTTTTTTATTGTGAGCTCTACTTGACTGCTCTACTCC
deletion F	TAACTTTTTTTTTTTTTTTTATTGTGAGCTCTACTTGACTGCTCTACTCC
distal	GGTGGTGGTGATTCTCATTTTACTTACCTTGAAGGTCCTAGTTCAGGTTG
proximal	TGCAGGGAACCTAAGGGACCTGGGTTGATGACGTGCTCTTCCAGAAAGAA
deletion F	TGCAGGGAACCTAAGGGACCTGGGTCTCTGAACTTTGTTGTGGAAATAAT
distal	ACCCTACTTTGAATATTAGCTGGATCTCTGAACTTTGTTGTGGAAATAAT
proximal	GTTGCTTTTGCCTCTTCCTGTTGTCCCAGAGAACCACCAGCCTGGGGATG
deletion F	TACTTCCAATACAACATGCATTTGCCAGGGACTTCCACTGCGCCTCTATG
distal	TACTTCCAATACAACATGCATTTGCCAGGGACTTCCACTGCGCCTCTATG
proximal	TGTGCCTTTATAGCAGCATGTTTTATAATCCTTTGGGTATATACCCAGTA
deletion G	TGTGCCTTTATAGCAGCATGTTTTATAATCCTTTGGGTATATACCCAGTA
distal	TGTGTCTTTATAGCAGCATGATTTATAATCCTTTGGGTATATACCCAGTA
proximal	ATGGGATCGCTGGGTCAAATGGTATTCCTAGTTCTAGATCCTTGAGGAAT
deletion G	ATGGGATCGCTGGGTCAAATGGTATTCCTAGTTCTAGATCCCTGAGGAAT
distal	ATGGGATTGCTGGGTCAAATGGTATTCCTAGTTCTAGATCCCTGAGGAAT
proximal	CACCACACTGTCTTCCACAATGGTTGAACTAGTTTACAGTCCCACCAACA
deletion G	CACCACACTGACTTCCACAATGGTTGAACTAGTTTACAGTCCCACCAACA
distal	CACCACACTGACTTCCACAATGGTTGAACTAGTTTACAGTCCCACCAACA
proximal	GGCCTCTCAAAGTGCTGGGATTACAGGTGTGAGTCACTGCATCTGACCTG
deletion H	GGCCTCTCAAAGTGCTGGGATTACAGGTGTGAGTCACTGCATCTGACCTG
distal	TTACAGGCGTGAGCCACCGCGCCCGGCCGCTTATTTTTACTCTTATTTAT
proximal	AGCTTTTTTTTTTGAGACGGAGTCTCACTCTGTCGCCTAGGTTTGAGTTA
deletion H	AGCTTTTTTTTTTGAGACGGAGTCTCACTCTATCACCCAGGCTGGAGTGC
distal	TTATTTATTTTTGGAGACTGAGTCTCACTCTATCACCCAGGCTGGAGTGC
proximal	AGTGGCACGATCTTGGCTCACTGCAACTTCCGCCTCCCGGGTTCAAGCCA
deletion H	AGTGGTGCGATCTTGGCTCACTGCAACCTCCACCTCCTGGGTTCAAGCGA
distal	AGTGGTGCGATCTTGGCTCACTGCAACCTCCACCTCCTGGGTTCAAGCGA
proximal	ATGCTCTGCACGCGTGTGTACGGCCCGTACGAGGCGGCCGGGCCCGCCAG
deletion 1	ATGCTCTGCACGCGTGTGTACGGCCCGTACGAGGCGGCCGGGCCCGCCAG
distal	CGGTACTCGCTCTGCGGCTGGGCTGGGAGATGACGAGGACCCCGGTGGGG
proximal	CCCCTTGACCACAGCGGCCGCGCCAGGGCTACCGGGGCCCGCGGCTGCAG
deletion 1	CCCCTTGACCACAGCGGCCGCGCCAGGAAGCTCGGGCCCCAGCGAGGAAA
distal	TCTGCCCGCACCCGGCCAAAGCCCAGGAAGCTCGGGCCCCAGCGAGGAAA
proximal	CCGCAGCTGCTGCAGCCGCTGCGGCTGCCGCCATCTGGCAGGAGGCATAG
deletion 1	GGCGCTCCAAGCCTCCTCGCGGCTTTCAGGTGAAAGAAAACGACTCCTTT
distal	GGCGCTCCAAGCCTCCTCGCGGCTTTCAGGTGAAAGAAAACGACTCCTTT
proximal	GAAAGGTTTTTTAAAACTCGGTTTCATACTATTATTATTACTAAGGACAA
deletion 2	GAAAGGTTTTTTAAAACTCGGTTTCATACTATTATTATTACTAAGGACAA
distal	GCCCAGAAACGGGTGTGACTGTACGAAGAAGCCTCGGCCTGGCCTGTCCC
proximal	CCGGGCAGGCTGAGGTCCCAACGTGGATGATCCGAGTTGGCCTCGCGCCG
deletion 2	CCGGGCAGGCTGAGGTCCCAACGTGGAATGGGGCAGGGGAGAGGATCTCT
distal	TCGCGCTCTCAGAGTGACTGGGCTGGAATGGGGCAGGGGAGAGGATCTCT
proximal	GGGCTCTGCAGCCACTGCCCTGTGCGCTCAGCACCTCTGGGGGCGATCAG
deletion 2	GGAAATAGTCGTCAGGGGCGCCGCCTGAATCACCTCTGCCTCTCCCTGCG
distal	GGAAATAGTCGTCAGGGGCGCCGCCTGAATCACCTCTGCCTCTCCCTGCG

```
proximal     TCTGGTTCTGGAAGCAGGAAACAGCTCTCAGGGTGATTCAGCCATGGGAA
deletion 3   TCTGGTTCTGGAAGCAGGAAACAGCTCTCAGGGTGATTCAGCCATGGGAA
distal       AATTACTAAGCACGAGGCAATCTAATGTGATTAAAAACAAATAATGAGAG

proximal     GAGCAGCATCCCTCATACCCCACCCTCATTCTTGCACCCAGCCAGGCAGG
deletion 3   GAGCAGCATCCCTCATACCCCACACAATTTAGCTATTAAAGTTAAAAAAT
distal       ATTTTACCTGTGGGGAAATTGAATCAATTTAGCTATTAAAGTTAAAAAAT

proximal     ACAGCTCCCTTCTATTTGCTGCCATCCCTCACCCTCTATAATTGCTTGAG
deletion 3   TTTCAAAACATAAATGATGGAATACCTTCCATGAACAAACTAGAAGTTTG
distal       TTTCAAAACATAAATGATGGAATACCTTCCATGAACAAACTAGAAGTTTG
```

```
proximal     TAATATTTGCAAATCATATATATCTGACAAGATATTAATATCCAGAATAT
deletion 5   TAATATTTGCAAATCATATATATCTGACAAGATATTAATATCCAGAATAT
distal       TCTTGGGACTATTTATCTACGCATACTATCTTCTTGGCCCATACCTTTTT

proximal     ATAAAGAACTCCTACAACTCAATAACAGAAAACAAAGTTTAAAAATCAGT
deletion 5   ATAAAGAACTCCTACAACTCAATAAGAAGGGGATAGATCATTGACTATCT
distal       TTTTTTTTTTTTTTTTTTTTTGGGAGAAGGGGATAGATCATTGACTATCT

proximal     ACAGGTCTAGAAAAGATAATTCTCCAAAAAACATACAAATGACCAACAAG
deletion 5   TTTCTGTAGCTTCTATGGATATTGGTGTATTAATATCTTGTATATTTTCC
distal       TTTCTGTAGCTTCTATGGATATTGGTGTATTAATATCTTGTATATTTTCC
```

```
proximal     CCAGTATCCTCTGAGATGACACTTATATCTCACAACAGGATGTAATAACT
deletion 6   CCAGTATCCTCTGAGATGACACTTATATCTCACAACAGGATGTAATAACT
distal       TGAAAAAACTCACAGGCTGGGCATGGTGGCTCACACCTGTAATCCCAGGA

proximal     GATGTTCAATTATTTTGTAAAATCCCAAAGACAGAGAAAAAAAACTGCGT
deletion 6   GATGTTCAATTATTTTGTAAAATCTCA-GAGGTCAGGAGATGGAGACCAT
distal       CTTTGGGAGGCCGAGGCGGGCGGATCATGAGGTCAGGAGATGGAGACCAT

proximal     TCTACATACTGTTTAATAAGGCAGAAAAGTATATACTATTCTCTTTCTTT
deletion 6   TCTGGCCAACATGGTGAAACCCCGTCTCTGCTAAAAATACAAAAATTAGC
distal       TCTGGCCAACATGGTGAAACCCCGTCTCTGCTAAAAATACAAAAATTAGC
```

```
proximal     AGAGAGAAGAAAAGAAAAGAAGCCAGTGTGCTGGCTCACACCTGTAATCC
deletion 7   AGAGAGAAGAAAAGAAAAGAAGCCAGTGTGCTGGCTCACACCTGTAATCC
distal       CAATTTGAAAAAACTCACAGGCTGGGCATGGTGGCTCACACCTGTAATCC

proximal     CAGCACTTTGGGAGGCAGAGGCGGGCGGCTCACGAGGTCAGGAGTTTGAG
deletion 7   CAGCACTTTGGGAGGCAGAGGCGGGCGGATCATGAGGTCAGGAGATGGAG
distal       CAGGACTTTGGGAGGCCGAGGCGGGCGGATCATGAGGTCAGGAGATGGAG

proximal     ACCAGCATGGCCAACATGGTGAAACCCTGTCTCTACTAAAGATACAAAAA
deletion 7   ACCATTCTGGCCAACATGGTGAAACCCCGTCTCTGCTAAAAATACAAAAA
distal       ACCATTCTGGCCAACATGGTGAAACCCCGTCTCTGCTAAAAATACAAAAA
```

```
proximal     GTTTTCTGACTTTTTAATGATTGCCATTCTAACTTGTGTGAGATGGTATC
deletion 8   GTTTTCTGACTTTTTAATGATTGCCATTCTAACTTGTGTGAGATGGTATC
distal       GTTTCCTGACTTTTTAATGATCGCCGTTCTAACTGGTGTGCAATGGTATC

proximal     TCATTGTGGTTTTGATTTGCATTTCTCTGATGGCCAGTGATGGTGAGCAT
deletion 8   TCATTGTGGTTTTGATTTGCATTTCTCTGATGGCCAGTGATGGTGAGCAT
distal       TCATTGTGGTTTTGATTTGCATTTCTCTGATGGCCAGTGATGGTGAGCAT

proximal     TTTTTCATATGTTTTTTGGCTGCATAAATGTCTTCTTTTGAGAAGTGTCT
deletion 8   TTTTTCATGTGTCTGTTGGCTGCATAAATGTCTTCTTTTGAGAAGTGTCT
distal       TTTTTCATGTGTCTGTTGGCTGCATAAATGTCTTCTTTTGAGAAGTGTCT
```

```
proximal     GAAGTGCAGTGGCACGATCTCTCCGCCTCCTGGGTTCACACCATTCTCCT
deletion 9   GAAGTGCAGTGGCACGATCTCTCCGCCTCCTGGGTTCACACCATTCTCCT
distal       CGATCTCGGCTCACTGCAAACTCCACCTCCCAGGTTCACGCCATTCTCCT

proximal     GCCTCAGCCTCCCGAGTAGCTGGGACTACAGGCGCCCACCACCACACCCA
deletion 9   GCCTCAGCCTCCCGAGTAGCTGGGACTACAGGAGCCCGCCACCAGGCCTG
distal       GCCTCAGCCTCCCGAGCAGCTGGGACTACAGGAGCCCGCCACCAGGCCTG

proximal     CCTAATTTTTTGTATTTTTAGTAGAGACGGGGTTTCACCATGTTAGCCAG
deletion 9   GCTAATTTTTTTGTATTTTTAGTAGAGATGGGGTTTCACCATGTTAGCC
distal       GCTAATTTTTTTGTATTTTTAGTAGAGATGGGGTTTCACCATGTTAGCC
```

```
proximal     ACTTAAAAGCTTCCATAGACATAATATTTATTTATTTTGGCATGTTTTTA
deletion 10  ACTTAAAAGCTTCCATAGACATAATATTTATTTATTTTGGCATGTTTTTA
distal       GATACCACCTCCAGATGTGGTACCCTCTAAAAATAGCAGGTGGAGTCATC

proximal     ATAGGAGTTTTCTGATCTGAACGTGTACAAAAGCAGCAGTACAGTGGTAT
deletion 10  ATAGGAGTTTTCTGATCTGAACGTGTCCCTCAGTGCCATCTGGAAGTGGA
distal       TCCCCTGGCTAATTGTTCTAAGTGGTCCCTCAGTGCCATCTGGAAGTGGA

proximal     TTCATCTGTTCTAAACCACATCCTTTTACTGAATGTTATTCCGGAAGCAT
deletion 10  TCTCCTCAGTGTACCAGGACATCGCCCCAATTATCTATGCAACGGCTCCT
distal       TCTCCTCAGTGTACCAGGACATCGCCCCAATTATCTATGCAACGGCTCCT
```

```
proximal     TGGGTCTCACTATGTTGCCCAGGGTGGTCTCCAACTCCTGGCCTCAAGGG
deletion 12  TGGGTCTCACTATGTTGCCCAGGGTGGTCTCCAACTCCTGGCCTCAAGGG
distal       GTGAGGAGTATCAAAGAATATGTGGACATATTTTAATAACACTACACTTA

proximal     ATCCTCCCACCTCAGCTATTCACAGTGCTGGGATTACAGGCATGAGCCAC
deletion 12  ATCCTCCCACCTCAGCTATTCACAGGTACAAAACCATGATGGATGGGTAA
distal       ATGAACCAGTATTTTATTCATGATGGTACAAAACCATGATGGATGGGTAA

proximal     CATGTCTGACCCAGATGACTTTCTATTCATAAAATAAGGGAATGGAATTG
deletion 12  GAAAAAAAAAATCTATTCCAATTGCAAGACAGTCCAATGGATTTTTAATG
distal       GAAAAAAAAAATCTATTCCAATTGCAAGACAGTCCAATGGATTTTTAATG
```

```
proximal     ATGCGATTCTCCTGCCTCAGCCTCCCGAGTAGCTGGGATTACAGGCATGC
deletion 13  ATGCGATTCTCCTGCCTCAGCCTCCCGAGTAGCTGGGATTACAGGCATGC
distal       CGATTCTCCTGCCTCAGCCTCCCAAGTAGCTGGGATTATTATAGGCATGC

proximal     GCCACCACTCCTGGCTAATTTTGTATTTTTAGTAGAGACGGGGTTTCTCC
deletion 13  GCCACCACTCCTGGCTAATTTTGTATTTTTAGTAGAGATGGGGTTTCTCC
distal       ACCATCACGCCCGGCTAATTTTGTATTTTTAGTAGAGATGGGGTTTCTCC

proximal     ATGTTGGTCAGGCTGGTCTCGAACTCCCAGCCTCAGGTGACCCACCCACC
deletion 13  ATGTTGGTCAGGCTGGTCTCGAACTCCTGACCTCAGGTGATCCACCTGCC
distal       ATGTTGGTCAGGCTGGTCTCGAACTCCTGACCTCAGGTGATCCACCTGCC


proximal     CCGAGTAGCTGGGATTACAGCTACTGTAATTACAGCTACTGCATGCCACC
deletion 14  CCGAGTAGCTGGGATTACAGCTACTGTAATTACAGCTACTGCATGCCACC
distal       TAATCTAAAATGTATCCACTGCCTTTTTTTGTTTTTATAACACTGACCTT

proximal     ATGCCCAGCTAATTTTTTTTTTTTTTTTTTTTTTTAAGTAGAGATGGGGTT
deletion 14  ATGCCCAGCTAATTTTTTTTTTTTTTTTTTTTTTTAAGATAGAGTCTCACT
distal       TTAAGTTATTGCTTTTTTTTTTTTTTTTTTTTTTTAAGATAGAGTCTCACT

proximal     TCATTATGTTGGCCAGGCTGGTCTTGAACTCCTGACCTTGTGATCCGCCC
deletion 14  GTGTCATCAAGGCTGGAGTGCAGTGGTATGATCTTGGTTCACCACAACCT
distal       GTGTCATCAAGGCTGGAGTGCAGTGGTATGATCTTGGTTCACCACAACCT


proximal     TGTTCAGTTATTCCAAACCTGCTATAACTATTCTCCCATAGCAAACTCCC
deletion 15  TGTTCAGTTATTCCAAACCTGCTATAACTATTCTCCCATAGCAAACTCCC
distal       GACTCAAGTCCCAGGCCCAAAAGACTTTGGCTTTAAACCAAAAGAAAGAA

proximal     TCAATTAAACTTGCTGGTTTGGGTTCTGTTTCCCTGATGGTTACAGATGT
deletion 15  TCAATTAAACTTGCTGGTTTGGGTTTTATCTCTCTGACATTTGCTGGGGC
distal       CCATGCATCACGGGTGCCTCAGACTTTATCTCTCTGACATTTGCTGGGGC

proximal     TATTTCTGTATTTGCCTTGCCCTCTTTCACAAGACTGGGAAGATCTCCTG
deletion 15  ACCTGTTCTGAGTTGTGGCTCTGGCTCGGAGTAGCTCTGGGGACAATTGG
distal       ACCTGTTCTGAGTTGTGGCTCTGGCTCGGAGTAGCTCTGGGGACAATTGG


proximal     ATAGTCCTTTGGGTATATACCCAGTAACGGGATGGCTGGGTCAAATGGTA
deletion 16  ATAGTCCTTTGGGTATATACCCAGTAACGGGATGGCTGGGTCAAATGGTA
distal       ATAGTCCTTTGGATATATACCCAGTAATGAGATGGCTGGGTCAAATGGTA

proximal     TTTCTAGTTCTAGATCCCTGAGGAATCACCACACTGACTTCCACAATGGT
deletion 16  TTTYTAGTTCTAGATCCCTGAGGAATCGCCACACTGACTTCCACAATGGT
distal       TTTCTAGTTCTAGATCCCTGACGAATCGCCACACTGACTTCCACAATGGT

proximal     TGAACTAGTTTACAGTCCCACCAACAGTGTAAAAGTGTTCCTATTTCTCC
deletion 16  TGAACTAGTTTACAGTCCCACCAACAGTGTAAAAGTGTTCCTGTTTCTCC
distal       TGAACTAGTTTACAGTCCCACCAACAGTGTAAAAGTGTTCCTGTTTCTCC
```
