# Supplementary material for: Microhomology-Mediated Mechanisms Underlie Non-Recurrent Disease-Causing Microdeletions of the FOXL2 Gene or Its Regulatory Domain
Source: PLoS Genet. 2013 Mar 14;9(3):e1003358. doi: 10.1371/journal.pgen.1003358 (PMC3597517; doi:10.1371/journal.pgen.1003358)
Supplement: Table S1 — The genomic location and gene content of the FOXL2 encompassing and regulatory deletions. (PDF) [file pgen.1003358.s004.pdf]

Table S1. The genomic location and gene content of the *FOXL2* encompassing and regulatory deletions

Patient code	Start (Hg19)	End (Hg19)	Size (kb)	Genes overlapping with or located closest to the proximal breakpoint	Genes overlapping with or located closest to the distal breakpoint	Genes located in deleted region
A	138949150	138956510	7	2.7 kb centromeric to *PISRT1*	4.1 kb telomeric to *PISRT1*	*PISRT1*
B	138912808	139012600	100	39 kb centromeric to *PISRT1*	50.3 kb centromeric to *MRPS22*	*PISRT1*
C	138867472	139048942	181	44.4 kb telomeric to *BPESC1*	13.9 kb centromeric to *MRPS22*	*PISRT1*
D	138479902	138662725	183	1.7 kb telomeric to *PIK3CB*	340 bp centromeric to *FOXL2*	-
E	138805920	139012140	206	38 kb centromeric to *BPESC1*	50.7 kb centromeric to *MRPS22*	*BPESC1, PISRT1*
F	138938973	139294473	356	12.9 kb centromeric to *PISRT1*	*NMNAT3*	*PISRT1,* ***MRPS22****, COPB2, RBP2, RBP1, NMNAT3* (partial)
G	138745991	140393036	1647	6.2 kb telomeric to *PRR23B*	3.8 kb centromeric to *TRIM42*	*PRR23C, BPESC1, PISRT1,* ***MRPS22****, COPB2, RBP2, RBP1, NMNAT3, CLSTN2*
H	138741281	141762242	3021	1.5 kb telomeric to *PRR23B*	*TFDP2*	*PRR23C, BPESC1, PISRT1,* ***MRPS22****, COPB2, RBP2, RBP1, NMNAT3, CLSTN2, TRIM42, SLC25A36, SPSB4, ACPL2, ZBTB38, RASA2, RNF7, GRK7, ATP1B3, TFDP2* (partial)
1	138664845	138666255	1	*FOXL2*	*C3orf72*	***FOXL2****, C3orf72* (partial)
2	138658319	138666527	8	4.7 kb centromeric to *FOXL2*	*C3orf72*	***FOXL2****, C3orf72* (partial)
3	138649686	138736058	86	13.4 kb centromeric to *FOXL2*	1.8 kb centromeric to *PRR23B*	***FOXL2****, C3orf72, PRR23A*
4	138661542	138786728	125	1.5 kb centromeric to *FOXL2*	23 kb telomeric from *PRR23C*	***FOXL2****, C3orf72, PRR23A, PRR23B, PRR23C*
5	138475828	138857284	381	*PIK3CB*	13.3 kb telomeric to *BPESC1*	*PIK3CB* (partial), ***FOXL2****, C3orf72, PRR23A, PRR23B, PRR23C, BPESC1*
6	138019964	139735421	1715	*NME9*	*CLSTN2*	*NME9* (partial), *MRAS, ESYT3, CEP70, FAIM, PIK3CB,* ***FOXL2****, C3orf72, PRR23A, PRR23B, PRR23C, BPESC1, PISRT1,* ***MRPS22****, COPB2, RBP2, RBP1, NMNAT3, CLSTN2* (partial)

Patient code	Start (Hg19)	End (Hg19)	Size (kb)	Genes overlapping with or closest to the proximal breakpoint	Genes overlapping with or closest to the distal breakpoint	Genes located in deleted region
7	137894385	139735424	1841	*DBR1*	*CLSTN2*	*DBR1* (partial), *ARMC8, NME9, MRAS, ESYT3, CEP70, FAIM, PIK3CB,* ***FOXL2****, C3orf72, PRR23A, PRR23B, PRR23C, BPESC1, PISRT1,* ***MRPS22****, COPB2, RBP2, RBP1, NMNAT3, CLSTN2* (partial)
8	136887871	138746237	1858	158 kb from *IL20RB*	6.5 kb telomeric to *PRR23B*	*SOX14, DZIP1L, CLDN18, A4GNT, DBR1, ARMC8, NME9, MRAS, ESYT3, CEP70, FAIM, PIK3CB,* ***FOXL2****, C3orf72, PRR23A, PRR23B*
9	138532388	140753510	2221	106 kb telomeric to *PIK3CB*	17.2 kb centromeric to *SPSB4*	***FOXL2****, C3orf72, PRR23A, PRR23B, PRR23C, BPESC1, PISRT1,* ***MRPS22****, COPB2, RBP2, RBP1, NMNAT3, CLSTN2, TRIM42, SLC25A36*
10	136604806	139000361	2396	*NCK1*	48 kb telomeric to *PISRT1*	*NCK1* (partial), *IL20RB, SOX14, DZIP1L, CLDN18, A4GNT, DBR1, ARMC8, NME9, MRAS, ESYT3, CEP70, FAIM, PIK3CB,* ***FOXL2****, C3orf72, PRR23A, PRR23B, PRR23C, BPESC1, PISRT1,*
11	136007507	138671922	2664	*PCCB*	*C3orf72*	***PCCB*** (partial), *STAG1, SLC35G2, NCK1, IL20RB, SOX14, DZIP1L, CLDN18, A4GNT, DBR1, ARMC8, NME9, MRAS, ESYT3, CEP70, FAIM, PIK3CB,* ***FOXL2****, C3orf72* (partial)
12	138134298	141034621	2900	9.9 kb telomeric to *MRAS*	21.1 kb telomeric to *ACPL2*	*ESYT3, CEP70, FAIM, PIK3CB,* ***FOXL2****, C3orf72, PRR23A, PRR23B, PRR23C, BPESC1, PISRT1,* ***MRPS22****, COPB2, RBP2, RBP1, NMNAT3, CLSTN2, TRIM42, SLC25A36, SPSB4, ACPL2*
13	138602856	142458004	3855	60.2 kb centromeric to *FOXL2*	*TRPC1*	***FOXL2****, C3orf72, PRR23A, PRR23B, PRR23C, BPESC1, PISRT1,* ***MRPS22****, COPB2, RBP2, RBP1, NMNAT3, CLSTN2, TRIM42, SLC25A36, SPSB4, ACPL2, ZBTB38, RASA2, RNF7, GRK7, ATP1B3, TFDP2, GK5, XRN1,* ***ATR****, PLS1, TRPC1* (partial)
14	137934887	142100431	4166	*ARMC8*	*XRN1*	***FOXL2****, C3orf72, PRR23A, PRR23B, PRR23C, BPESC1, PISRT1,* ***MRPS22****, COPB2, RBP2, RBP1, NMNAT3, CLSTN2, TRIM42, SLC25A36, SPSB4, ACPL2, ZBTB38, RASA2, RNF7, GRK7, ATP1B3, TFDP2, GK5, XRN1* (partial)
15	135099979	139713853	4614	120 kb telomeric to *EPHB1*	*CLSTN2*	*PPP2R3A, MSL2,* ***PCCB****, STAG1, SLC35G2, NCK1, IL20RB, SOX14, DZIP1L, CLDN18, A4GNT, DBR1, ARMC8, NME9, MRAS, ESYT3, CEP70, FAIM, PIK3CB,* ***FOXL2****, C3orf72, PRR23A, PRR23B, PRR23C, BPESC1, PISRT1,* ***MRPS22****, COPB2, RBP2, RBP1, NMNAT3, CLSTN2* (partial)
16	136887730	142397685	5510	158 kb telomeric to *IL20RB*	*PLS1*	*SOX14, DZIP1L, CLDN18, A4GNT, DBR1, ARMC8, NME9, MRAS, ESYT3, CEP70, FAIM, PIK3CB,* ***FOXL2****, C3orf72, PRR23A, PRR23B, PRR23C, BPESC1, PISRT1,* ***MRPS22****, COPB2, RBP2, RBP1, NMNAT3, CLSTN2, TRIM42, SLC25A36, SPSB4, ACPL2, ZBTB38, RASA2, RNF7, GRK7, ATP1B3, TFDP2, GK5, XRN1,* ***ATR****, PLS1* (partial)

Disease-associated genes according to Online Mendelian Inheritance in Man (OMIM) are shown in bold. Their corresponding OMIM numbers are: *PCCB* (606054), *FOXL2* (110100), *MRPS22* (611719) and *ATR* (614564).
