# Supplementary material for: Microhomology-Mediated Mechanisms Underlie Non-Recurrent Disease-Causing Microdeletions of the FOXL2 Gene or Its Regulatory Domain
Source: PLoS Genet. 2013 Mar 14;9(3):e1003358. doi: 10.1371/journal.pgen.1003358 (PMC3597517; doi:10.1371/journal.pgen.1003358)
Supplement: Table S2 — Overview of sequence motifs. (PDF) [file pgen.1003358.s005.pdf]

Table S2. Overview of sequence motifs

Motif name	Motif sequence	Number of sequences	
		Random control population (n=500)	Observed breakpoint regions (n=48)
X-element *E. coli*	GCTGGTGG	1	1
Ade6-M26	ATGACGT	-	1
ARS consensus *S. cerevisiae*	WTTTATRTTTW	4	1
ARS consensus *S. Pombe*	WRTTTATTTAW	3	2
Consensus SAR 1	AATAAAYAAA	5	2
Consensus SAR 2	TTWTWTTWTT	46	7
Consensus SAR 3	WADAWAYAWW	109	14
Consensus SAR 4	TWWTDTTWWW	120	10
Deletion hotspot consensus	TGRRKM	385	31
DNA polymerase arrest site	WGGAG	285	24
DNA polymerase a frameshift hotspot 1	TCCCCC	32	1
DNA polymerase a frameshift hotspot 2	CTGGCG	7	-
DNA polymerase b frameshift hotspot 1	ACCCWR	138	17
DNA polymerase a/b frameshift hotspot 1	ACCCCA	42	7
DNA polymerase a/b frameshift hotspot 2	TGGNGT	142	15
D. Topoisomerase 2 consensus	GTNWAYATTNATNNR	2	-
Heptamer recombination signal	CACAGTG	23	3
Human hypervariable minisatelliets sequence 1	GGAGGTGGGCAGGARG	-	-
Human hypervariable minisatelliets sequence 2	AGAGGTGGGCAGGTGG	-	-
Human minisatelliets core sequence	GGGCAGGARG	1	-
Human replication origin consensus	WAWTTDDWWWDHWGWHMAWTTDHWGWHMAWTT	-	-
Human minisatellites conserved sequence/X-like element	GCWGGWGG	17	3
Ig heavy chain class switch repeat 1	GAGCT	116	6
Ig heavy chain class switch repeat 2	GGGCT	99	10
Ig heavy chain class switch repeat 3	GGGGT	87	12
Ig heavy chain class switch repeat 4	TGGGG	134	15
Ig heavy chain class switch repeat 5	TGAGC	130	15
LTR-IS motif	TGGAAATCCCC	-	-
Mariner transposon-like element	GAAAATGAAGCTATTTACCCAGGA	-	-
Murine MHC recombination hotspot	CAGRCAGR	25	2
Murine parvovirus recombination hotspot	CTWTTY	239	20
Nonamer recombination signal	ACAAAAACC	2	-
Pur-binding site	GGNNGAGGGAGARRRR	-	-
Recombination hotspot	CCNCCNTNNCCNC	10	-
Retrotransposon	TCATACACCACGCAGGGGTAGAGGACT	-	-
Translin-binding site 1	ATGCAG	34	5
Translin-binding site 2	GCCCWSSW	47	4
Vaccinia topoisomerase I consensus	YCCTT	257	16
Vaccinia topoisomerase II consensus	RNYNNCNNGYNGKTNYNY	2	-
XY32 homopurine-pyrimidine H-palindrome motif	AAGGGAGAARGGGTATAGGGRAAGAGGGAA	-	-
