# Supplementary material for: Microhomology-Mediated Mechanisms Underlie Non-Recurrent Disease-Causing Microdeletions of the FOXL2 Gene or Its Regulatory Domain
Source: PLoS Genet. 2013 Mar 14;9(3):e1003358. doi: 10.1371/journal.pgen.1003358 (PMC3597517; doi:10.1371/journal.pgen.1003358)
Supplement: Table S3 — Sequences of non-B DNA conformations. (PDF) [file pgen.1003358.s006.pdf]

Table S3. Sequences of non-B DNA conformations

Deletion breakpoint	[(YR•YR)$_n$] repeat	Direct repeat	Oligo(G)$_n$ tract
H (proximal)	-	TCACTGCA	-
1 (proximal)	CTCTGCACGCGTGTGTACGG	GGGCCCGC	**GG**CCGCGCCA**GG**GCTACC**GG**GGCCCGC**GG**
1 (distal)	-	-	**GGA**AGCTCG**GG**CCCCAGCGA**GG**AAA**GG**
2 (proximal)	-	-	**GG**ATGATCCGAGTT**GG**CCTCGCGCC**GGGG**
2 (distal)	-	-	**GG**AATG**GG**GCAG**GG**GAGA**GG**
3 (proximal)	-	CATCCCTCA	-
5 (distal)	-	ACTATCTT	-
6 (distal)	-	-	**GG**ATCATGA**GG**TCA**GG**AGAT**GG**
7 (proximal)	-	-	**GG**GA**GG**CAGA**GG**CG**GG**
7 (distal)	-	-	**GG**GA**GG**CCGA**GG**CG**GG**, **GG**ATCATGA**GG**TCA**GG**AGAT**GG**
9 (distal)	-	-	**GG**GACTACA**GG**AGCCCGCCACCA**GG**CCT**GG**
11 (distal)	-	-	**GG**CT**GG**AGTGCAGT**GG**CATGAACTT**GG**
14 (proximal)	-	TTTTTTTTTTT	-
14 (distal)	-	TTTTTTTTTTT, CTTTTTTT	-
