# Supplementary material for: Microhomology-Mediated Mechanisms Underlie Non-Recurrent Disease-Causing Microdeletions of the FOXL2 Gene or Its Regulatory Domain
Source: PLoS Genet. 2013 Mar 14;9(3):e1003358. doi: 10.1371/journal.pgen.1003358 (PMC3597517; doi:10.1371/journal.pgen.1003358)
Supplement: Table S4 — The presence of microhomology and the most likely molecular mechanism in previous studies. (PDF) [file pgen.1003358.s007.pdf]

Table S4. The presence of microhomology and the most likely molecular mechanism in previous studies

Study	Locus	Associated disease	Junctions analyzed	Percentage micro-homology	Most likely molecular mechanism
Nobile (2002)	Xp21.2 - Xp21.1	Duchenne muscular dystrophy	5	60%	NHEJ
Toffolatti (2002)	Xp21.2 - Xp21.1	Duchenne muscular dystrophy	14	50%	NHEJ
Inoue (2002)	Xq22	Pelizaeus-Merzbacher	3	66.7%	1/3 *Alu-Alu* NAHR, 2/3 NHEJ
Venturin (2004)	17q11.2	Neurofibromatosis 1	1	-	NHEJ
Shaw (2005)	17q11.2	Smith-Magenis syndrome	4	75%	2/4 *Alu-Alu* NAHR, 2/4 NHEJ
